# PReS13-SPK-1332: Assessment of disease activity and damage in juvenile idiopathic arthritis

**DOI:** 10.1186/1546-0096-11-S2-I21

**Published:** 2013-12-05

**Authors:** A Ravelli

**Affiliations:** 1 Istituto Giannina Gaslini, Università degli Studi di Genova, Genova, Italy

## 

A vast array of instruments are available for measuring disease activity in juvenile idiopathic arthritis (JIA). However, due to the high variability in the clinical presentation and course of JIA, no single measure can reliably capture disease activity in all patients. On the other hand, assessment of all measures individually may cause methodological and statistical problems, especially when these measures are employed as endpoints in clinical trials. Several approaches can be followed to achieve a more rational and standardized evaluation. One of these approaches is based on the so-called composite disease activity scores, which are made of a pool of individual measures and are aimed to quantify the absolute level of disease activity by providing one summary number on a continuous scale. Recently, the first composite disease activity score for JIA, named Juvenile Arthritis Disease Activity Score (JADAS), has been developed. In validation analyses, it was found to have good metrologic properties, including the ability to predict the disease outcome. The cutoff values of the JADAS that corresponded with the states of inactive disease and minimal disease activity, or reflected the physician's, parent's or child's subjective rating of remission or the parent's or child's satisfaction with the outcome of the illness were established recently. These cutoffs represent an additional clinical tool that, if applied regularly in daily practice, may allow tighter control of therapy, support the optimization of treatment on an individual patient basis, and help prevent the development of joint damage and physical disability. JIA is characterized by prolonged synovial inflammation that may cause irreversible alterations in joint structures. Permanent changes may also occur in extra-articular organs/systems, such as the eye (as a complication of chronic anterior uveitis), or may result from side effects of medications. The Juvenile Arthritis Damage Index (JADI) was devised to enable a thorough detection of articular and extra-articular damage in children with JIA. The JADI is aimed to capture damage, defined as persistent changes in anatomy, physiology, pathology or function, which may be the consequence of previous active disease, side effects of therapy, or co-morbid conditions, is not due to currently active arthritis, and is present for at least 6 months. Damage is often irreversible and cumulative and, thus, damage scores are most frequently expected to increase or remain stable over time. However, because some forms of damage may improve or even resolve in growing children, in some cases scores may decrease. The index is composed of two parts, one devoted to the assessment of articular damage (JADI-A) and one devoted to the assessment of extraarticular damage (JADI-E). In validation analyses, the JADI was found to be feasible and to possess both face and content validity; furthermore, it exhibited good convergent construct validity, excellent reliability (interrater agreement and internal consistency), and strong discriminative validity in a large cohort of JIA patients with long-standing disease.

## Disclosure of interest

None declared.

